# Comparing ChatGPT3.5 and Bard recommendations for colonoscopy intervals: Bridging the gap in healthcare settings

**DOI:** 10.1055/a-2586-5912

**Published:** 2025-06-17

**Authors:** Maziar Amini, Patrick W. Chang, Rio O. Davis, Denis D. Nguyen, Jennifer L Dodge, Jennifer Phan, James Buxbaum, Ara Sahakian

**Affiliations:** 1 12223 Division of Gastrointestinal and Liver Diseases, University of Southern California Keck School of Medicine, Los Angeles, United States; 2 12223 Department of Internal Medicine, Division of Gastroenterology, University of Southern California Keck School of Medicine, Los Angeles, United States; 3 5783 School of Science and Engineering, Tulane University, New Orleans, United States; 4 5116 Division of Gastrointestinal and Liver Diseases, University of Southern California, Los Angeles, United States; 5 Medicine/Gastroenterology, University of California, San Francisco, San Francisco, United States

**Keywords:** Endoscopy Lower GI Tract, CRC screening, Quality and logistical aspects, Image and data processing, documentatiton, Epidemiology

## Abstract

**Background and study aims:**

Colorectal cancer is a leading cause of cancer-related deaths, with screening and surveillance colonoscopy playing a crucial role in early detection. This study examined the efficacy of two freely available large language models (LLMs), GPT3.5 and Bard, in recommending colonoscopy intervals in diverse healthcare settings.

**Patients and methods:**

A cross-sectional study was conducted using data from routine colonoscopies at a large safety-net and a private tertiary hospital. GPT3.5 and Bard were tasked with recommending screening intervals based on colonoscopy reports and pathology data and their accuracy and inter-rater reliability were compared to a guideline-directed endoscopist panel.

**Results:**

Of 549 colonoscopies analyzed (N = 268 at safety-net and N = 281 private hospital), GPT3.5 showed better concordance with guideline recommendations (GPT3.5: 60.4% vs. Bard: 50.0%, *P* < 0.001). In the safety-net hospital, GPT3.5 had a 60.5% concordance rate with the panel compared with Bard’s 45.7% ( *P* < 0.001). For the private hospital, concordance was 60.3% for GPT3.5 and 54.3% for Bard ( *P* = 0.13). GPT3.5 showed fair agreement with the panel (kappa = 0.324), whereas Bard displayed lower agreement (kappa = 0.219). For the safety-net hospital, GPT3.5 showed fair agreement with the panel (kappa = 0.340) whereas Bard showed slight agreement (kappa = 0.148). For the private hospital, both GPT3.5 and Bard demonstrated fair agreement with the panel (kappa = 0.295 and 0.282, respectively).

**Conclusions:**

This study highlights the limitations of freely available LLMs in assisting colonoscopy screening recommendations. Although the potential of freely available LLMs to offer uniformity is significant, the low accuracy, as noted, excludes their use as the sole agent in providing recommendations.

## Introduction

Colorectal cancer (CRC) ranks as the second leading cause of cancer-associated mortality in the United States 1 . Screening colonoscopy reduces incidence of colon cancer 2 . Screening guidelines are continuously updated, with recent updates now recommending screening beginning at age 45 3 .

Artificial intelligence (AI) is an area of great interest in gastroenterology, with many projects aiming to improve detection of precancerous legions 4 . While promising, a common thread among these AI systems is their reliance on machine learning, which necessitates extensive datasets for model training 5 . Specifically, in the realm of colonoscopy screening intervals, previous studies have used machine learning and natural language processing to successfully predict surveillance screening intervals with high concordance compared to endoscopists; however, they were reliant on a large volume of initial training data 6 7 . Large language models (LLMs) have been a recent area of interest, with their transformer architecture unlocking the potential for training on a broad set of data and performing tasks without specifically being trained for them 8 .

Public access to free, commercially available chat bots has emerged for access to these models. These include Chat Generative Pre-Trained Transformers (ChatGPT) by OpenAI (OpenAI LLC, San Francisco, California, United States) in conjunction with Microsoft (Microsoft Corporation, Redmond, Washington, United States) and Bard developed by Google (Alphabet inc., Mountainview, California, United States). Both models are not only freely accessible but have also found applications in gastroenterology, and have previously been explored to generate medical responses for patients with nonalcoholic fatty liver disease, answering questions about colonoscopy, cirrhosis, and hepatocellular carcinoma, and triaging admissions for ulcerative colitis patient presentations 9 10 11 12 .

Yet, the collaboration of AI and gastroenterology is not without its challenges. There are several previously cited concerns that involve potential differences in performance of AI depending on patient socioeconomic status or insurance type 13 14 . Socioeconomic status is of particular relevance and has consistently demonstrated worse CRC outcomes, with factors including worsening screening rates and unequal early stage detection 15 .

In our previous work with ChatGPT4, we demonstrated use of large language models (LLMs) for predicting colonoscopy screening intervals as a proof of concept 16 . However, in this cross-sectional study, we explored use of GPT3.5 and Bard, two free, publicly available LLMs, as potential second readers of routine colonoscopy results to generate screening interval recommendations in a safety-net versus tertiary private hospital setting. We aimed to assess accuracy and inter-rater reliability of screening recommendations generated by GPT3.5 and Bard versus a guideline-directed endoscopist panel and compare performance in two distinct healthcare settings: a safety-net hospital and a tertiary private medical institution.

## Patients and methods

### Design and setting

We performed a cross-sectional study using colonoscopy data from a major safety-net hospital with a largely uninsured population, Los Angeles General Medical Center, and a large, private tertiary referral center, Keck Medical Center of University of Southern California, in Los Angeles, California, United States.

### Participants

From January to May 2023, consecutive adult patients (aged > 18 years) undergoing routine colonoscopy at the two institutions were selected for study inclusion. Routine colonoscopies were defined as routine screening or surveillance colonoscopies, work-up for anemia, indeterminate imaging findings, other patient symptoms (i.e. change in bowel habits), or other appropriate complaints after clinical work-up. A summary of patient characteristics is included in Table 1 .

**Table:** **Table 1** Characteristics of clinical history, colonoscopy data, and biopsy results for patients evaluated by ChatGPT 3.5 and Bard.

	Patient characteristics	Safety net hospital	Tertiary private hospital	P value (Safety net vs private)
**Demographic data**				
	Total N	268	281	
	Age (mean ± SD)	57.5 ± 8.6	59.7 ± 9.7	0.01*
	Female – N (%)	155 (57.8)	142 (50.5)	0.09
	Family history of colon cancer – N (%)	25 (9.3)	14 (5.0)	0.05*
**Endoscopic findings per procedure**				
	Polypectomy performed - N (%)	194 (72.4)	162 (57.7)	< 0.001*
	Total polyps found (all patients, Mean ± SD)	2.1 ± 2.4	1.5 ± 2.0	0.001*
	Largest polyp size in mm (all patients, mean ± SD)	4.2 ± 3.1	4.4 ± 3.4	0.62
**Pathology**				
Hyperplastic	Total found (range)	126 (0–16)	144 (0–10)	
	Number with polyp (%)	56 (20.9)	92 (32.7)	
	Average number (along with finding, mean ± SD)	2.3 (2.2)	1.6 (1.4)	0.02*
Tubular adenoma	Total found (range)	259 (0–10)	200 (0–8)	
	Number with finding (%)	129 (48.1)	98 (34.9)	
	Average number (along with finding, mean ± SD)	2.01 (1.4)	2.04 (1.5)	0.87
	Mean size, mm (along with finding, mean ± SD)	4.40 (2.7)	5.03 (3.8)	0.14
	Number < 6 mm (along with finding)	101	65	
	6–9 mm	20	20	
	> 9 mm	8	13	
Tubulovillous	Total found (Range)	10 (0–2)	7 (0–2)	
	Number with finding (%)	9 (3.4)	6 (2.1)	
	Average number (along with finding, Mean ± SD)	1.1 (0.3)	1.2 (0.4)	0.78
	Mean size, mm (along with findings, Mean ± SD)	9.0 (3.6)	7.7 (3.9)	0.64
	Number <6mm (along with finding)	1	2	
	6–9mm	0	2	
	> 9 mm	2	2	
Sessile Serrated Lesions	Total found (range)	0 (0)	9 (0–2)	
	Number with finding (%)	0 (0)	8 (2.8)	
	Average number (along with finding, Mean ± SD)	0 (0)	1.1 (0.4)	N/A
	Mean size, mm (along with findings, Mean ± SD)	0 (0)	4.9 (3.0)	N/A
	Number < 6 mm (along with finding)	N/A	6	
	6–9 mm	N/A	1	
	> 9 mm	N/A	1	
*Agreement beyond chance, P < 0.05. SD, standard deviation.				

Exclusion criteria included patients with colon cancer, age < 45 years, inflammatory bowel disease (IBD), genetic polyposis syndromes, referrals for advanced therapeutic procedures, overt gastrointestinal bleeding, colonoscopy with inadequate bowel preparation, and procedures with inadequate patient data. This was to capture a low- to moderate-risk population in which use of modern AI may impact general clinical practice. To determine the appropriate sample size for this study, we aimed to detect a minimum 10% difference in accuracy of colonoscopy surveillance recommendations between the two LLMs. Based on a prior study 16 , we anticipated a 40% exclusion rate due to our criteria and calculated that 395 patients would be required to achieve 80% power at a significance level of 0.05. To account for potential variability and limited prior data on LLM comparisons in this domain, we conservatively increased the sample size to 1,000 patients to ensure sufficient statistical power and robustness of our results.

### Data collection

Patient demographics (age, gender, family history of colon cancer) and clinical data (procedure and pathology reports) were collected as deidentified text via the respective hospital electronic medical record (EMR) systems. Data were specifically captured verbatim from the following EMR documents: pre-procedure history and physical, family history, colonoscopy procedure report findings, and pathology report findings. In cases without a polypectomy, biopsy pathology results were input with “no path”. Any identifying patient information was removed manually prior to entering the data into the AI engine.

Emphasis was placed on using unaltered reports from the gastroenterologists to mimic the real-world implication of using AI. For instances in which the history and physical or family history text fields were not entered or left incomplete by the clinician, text was left unaltered. Prior to study initiation, we secured Institutional Review Board approval at University of Southern California for a strategy to de-identify all clinical data used in this study. We followed the Health Insurance Portability and Accountability Act Privacy Rule de-identification standard by removing information associated with the following predetermined 18 categories including: 1) names; 2) geographic references smaller than a state; 3) personal dates except year; 4) telephone number; 5) vehicle license numbers; 6) fax numbers; 7) device identifiers; 8) emails; 9) universal resource locators; 10) Social Security numbers; 11) internet protocol address; 12) medical record numbers; 13) biometric identifiers; 14) health plan beneficiary numbers; 15) full face photographs; 16) personal account numbers; 17) unique personal codes; and 18) personal certificates) and removing any other possible information that might identify a patient.

### Artificial intelligence

Two LLMs were used in this study: Generative Pre-trained Transformer Version3.5 (ChatGPT3.5) turbo by OpenAI (San Francisco, California, United States) 17 and Bard by Google (Mountain View, California, United States) 18 .

GPT3.5 is a LLM trained on massive amounts of text data sourced from the internet, allowing it to learn from a diverse range of topics and styles. It is an iterative improvement on the previously released GPT3. The exact contents of its dataset are proprietary, but the transformer model is known to consist of approximately 154 billion parameters 19 . The data set consists of large amounts of scraped web data, books, and Wikipedia articles, and is notably not pretrained for gastroenterology applications 19 . Notably, GPT3.5-turbo was the specific version used in this study and has costs significantly less to operate than previous iterations. GPT3.5 was the underlying model selected within the ChatGPT platform for this study, allowing for textual interaction with the model. GPT3.5 persists memory from previous conversations, which can be useful for users to interact with prior responses. However, for the purposes of this study, a new chat session was initiated for every input to ensure a standardized response with no memory of previous answers.

Bard is a generative AI chat bot powered by Google PaLM 2 20 . Similar to GPT3.5, the underlying architecture is a transformer model, trained broadly on web documents, books, code, mathematics, and conversational data 21 . The number of parameters trained by the dataset is approximately 340 billion 22 , thus approximately twice the size of OpenAI GPT3.5. As done with input provided to ChatGPT, a new chat session was initiated for each input to ensure a standardized response.

Both AI models were initialized with a standardized prompt requesting the model to provide the appropriate screening interval based on the latest 2020 US Multi-Society Task Force (USMSTF) on Colorectal Cancer Guidelines supported by the American Society for Gastrointestinal Endoscopy, American Gastroenterology Association, and American College of Gastroenterology 23 . Prompt engineering was conducted through iterative testing on a diverse set of 10 sample cases, chosen to represent the range and complexity of cases likely to appear in real-world scenarios. Each prompt iteration was rigorously tested across all 10 cases to ensure it consistently produced the desired results, accounting for nuances and variability typically found in medical reports. This process involved refining the prompt until we achieved reliable, reproducible outcomes across the sample, ensuring robustness in response to variations within this representative set of cases. This was subsequently followed by a sentence to specifically give a recommendation interval and to avoid summarizing the report. This was then followed by pre-procedure history and physical, any relevant family history of colon cancer, the colonoscopy procedure report, and the pathology report, all pasted in tandem. ChatGPT3.5 and Bard complete responses and recommended interval were recorded for later comparison ( **Supplementary Table 1** ).

### Reference standard

The reference standard was based on the USMSTF guidelines for colonoscopy screening intervals, subsequently referred to as the “guideline-panel”. Utilizing the approach of earlier studies which assessed AI in setting colonoscopy intervals, both the second and senior authors (third-year gastroenterology fellow and gastroenterology attending) separately assessed and gave recommendations in line with the recent USMSTF guidelines for all colonoscopies. Any differing views between the authors were settled by consulting the second-most senior author. A consensus was characterized as advice from both parties that either matched numerically or fell entirely within the guideline range. After this process was completed, 10% of the overall sample was sent to the second-senior study author to audit the validity reference standard and find 95% agreement with our guideline panel. Our procedure mirrored panels previously used in both theoretical studies for LLM and NLP AI types 6 24 .

### Outcomes

The primary outcome was accuracy of screening recommendations, as defined as the percentage of screening intervals (for surveillance and rescreening) provided by the LLM, matching the gold-standard panel guideline recommendations. Secondary outcomes were inter-rater reliability between the LLM and gold standard guideline panel recommendations. All colonoscopy intervals within the USMSTF guidelines are shown in Table 2 , with all other miscellaneous recommendations represented by “other.” These included various non-specific recommendations such as “6 months” or “3–10 years.” Instances in which the LLM deferred a recommendation to the gastroenterologist are included under “no answer.”

**Table:** **Table 2** Summary of intervals recommended by guideline panel, GPT3.5, and Bard.

Recommendation	Guideline Panel	GPT3.5	Bard
10 years	273 (49.7%)	248 (45.2%)	176 (32.1%)
7–10 years	148 (27.0%)	69 (12.6%)	132 (24.0%)
5–10 years	4 (0.7%)	91 (16.6%)	26 (4.7%)
5 years	34 (6.2%)	39 (7.1%)	90 (16.4%)
3–5 years	43 (7.8%)	39 (7.1%)	20 (3.6%)
3 years	47 (8.6%)	44 (8.0%)	92 (16.8%)
1 year	0 (0%)	0 (0.0%)	0 (0.0%)
Other interval	0 (0%)	13 (2.4%)	9 (1.6%)
No answer	0 (0%)	6 (1.1%)	4 (0.7%)

### Statistical analysis

To characterize the study population, means with standard deviations (SDs) and frequency with percent were calculated, with further stratification by safety-net and tertiary private hospital. For each characteristic, either an independent samples *t* -test (for continuous variables) or a chi-square test (for binary variables) was conducted to compare sample differences between the safety-net hospital and the tertiary private hospital.

All intervals suggested by the LLMs were graded as either concordant or discordant when compared with the guideline panel. Specifically, if an interval suggested by the LLM fell within range of the guideline panel, it was marked as concordant, whereas any interval that was out of range or exceeded the range suggested by the guideline panel was marked as discordant. We used McNemar’s test of proportion to compare error rates, defined as discordant outcomes, between GPT3.5 and Bard, with a further subanalysis comparing the same error rates for the safety-net and tertiary private hospitals. We used Fleiss’ kappa statistic to measure overall inter-rater reliability of follow-up screening recommendations of both LLMs compared with the guideline panel and for each guideline panel recommendation interval individually. Deference to the gastroenterologist was excluded from this analysis. Subanalyses were performed using the same methodology to assess inter-rater reliability separately for the safety-net and private hospitals.

We computed the mean colonoscopy interval for each LLM. To facilitate this, intervals were transformed into numerical values by taking their average; for instance, "7–10" was represented as 8.5. Subsequently, these mean intervals were compared using paired samples *t* -test.

To explore specific areas in which the LLMs differ in their recommendations, patient demographic and clinical characteristics were described by concordant and discordant intervals within each LLM to guideline comparison. Discordant intervals were further categorized as either earlier or later than those suggested by the guideline panel.

We based our analysis on accepted study design that evaluates correlation among raters 25 26 27 . Using traditional definitions, two standards for inter-rater reliability for kappa value were included as reference: Landis and Koch's classification of Kappa: < 0 poor agreement, 0.00–0.20 slight agreement, 0.21–0.40 fair agreement, 0.41–0.6 moderate agreement, 0.61–0.8 substantial agreement, 0.81–1.00 almost perfect agreement 28 . This study was reviewed and approved by the Institutional Review Board at University of Southern California.

## Results

**Fig. 1:**
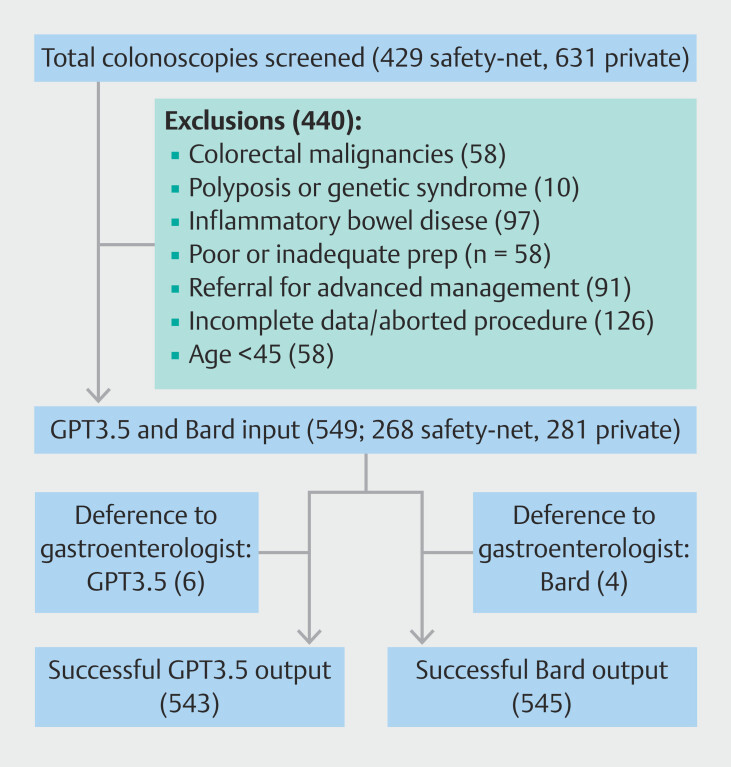
Flow of screening and patient capture in 549 deidentified data input into Bard and GPT3.5.

From January to May 2023, 549 colonoscopies met the criteria for analysis. A total of 440 cases were excluded ( Fig. 1 ). Table 1 lists detailed study population characteristics, including differences between cohort characteristics.

**Fig. 2:**
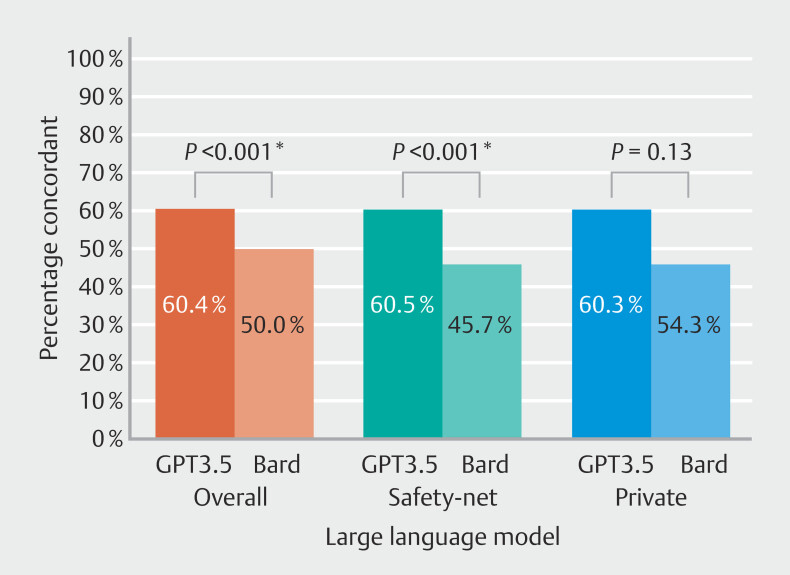
Percentage concordant for GPT3.5 and Bard vs AGA guideline-directed panel by hospital type.

Overall ( Fig. 2 ), guideline panel recommendations agreed with GPT3.5 in 60.4% of cases, compared with 50.0% for Bard ( *P* < 0.001). Within the safety-net hospital patients, guideline panel recommendations agreed with GPT3.5 in 60.5% of cases, compared with 45.7% for Bard ( *P* < 0.001). For private hospital patients, guideline-driven endoscopist panel recommendations agreed with GPT3.5 in 60.3% of cases, compared with 54.3% for Bard ( *P* = 0.13).

Overall ( Table 3 ), inter-rater reliability for GPT3.5 versus the guideline panel showed fair agreement, and agreement beyond chance (Fleiss’ kappa = 0.324; 95% confidence interval [CI] 0.280–0.368) corresponding to 60.4% concordant, 28.7% discordant early screening, and 10.9% discordant late screening. For Bard, the overall inter-rater reliability compared with the guideline-panel showed fair agreement (Kappa = 0.219; 95% CI 0.174–0.264) corresponding to 50.0% concordant, 38.2% discordant earlier screening, and 11.7% discordant later screening. Notably, overall inter-rater reliability was higher for GPT3.5 compared with Bard with non-overlapping CIs.

**Table:** **Table 3** Inter-rater reliability for overall agreement and by interval of recommendation for GPT3.5 vs. guideline panel and Bard vs guideline panel by Fleiss Kappa test.

Inter-rater reliability	Guideline panel vs. GPT3.5: Overall	Guideline panel vs. Bard: Overall
Overall	0.324 (0.280–0.368)*	0.219 (0.174–0.264)*
Recommended follow-up (yrs)		
10	0.587 (0.504–0.671)*	0.401 (0.317–0.485)*
7–10	0.190 (0.107–0.274)*	0.243 (0.159–0.326)*
5–10	–0.095 (–0.178 to–0.011)	0.040 (–0.043–0.124)
5	0.398 (0.315–0.482)*	0.091 (0.007–0.175)*
3–5	0.130 (0.047–0.214)*	–0.027 (–0.111–0.056)
3	0.365 (0.281–0.449)*	0.086 (0.002–0.169)*
1	N/A	N/A
Other interval	–0.012 (–0.096–0.072)	–0.008 (–0.092–0.075)
*Agreement beyond chance, P < 0.05.		

**Fig. 3:**
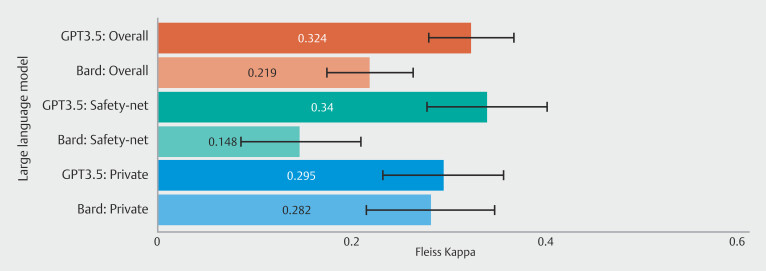
Inter-rater reliability for GPT3.5 and Bard vs AGA guideline-directed panel by hospital type.

For GPT3.5 ( Table 4 Fig. 3 ), agreement was fair in both the safety-net and private hospital settings with similar inter-rater reliability (kappa = 0.340, 95% CI 0.279–0.402 vs kappa = 0.295, 95% CI 0.232–0.357), corresponding to 61% and 60% concordance respectively ( *P* = 0.96). Discordance with early screening and later screening compared with the guideline panel accounted for 27% and 12% of observations in the safety-net setting compared with 30% and 10% for the private hospital.

**Table:** **Table 4** Inter-rater reliability by overall agreement and by interval of recommendation for GPT3.5 vs. AGA guidelines and Bard vs AGA guidelines by Fleiss Kappa test, stratified by hospital.

Inter-rater reliability	Guideline panel vs GPT3.5: Safety-net hospital	Guideline panel vs Bard: Safety-net hospital	Guideline panel vs GPT3.5: Private hospital	Guideline panel vs Bard: Private hospital
Overall	0.340 (0.278–0.402)*	0.148 (0.086–0.210)*	0.295 (0.232–0.357)*	0.282 (0.216–0.348)*
Recommended follow-up (yrs)				
10	0.584 (0.464–0.703)*	0.305 (0.185–0.425)*	0.578 (0.461–0.695)*	0.464 (0.348–0.581)*
7–10	0.179 (0.059–0.299)*	0.169 (0.049–0.288)*	0.186 (0.069–0.303)*	0.315 (0.198–0.432)*
5–10	–0.083 (–0.203–0.037)	–0.023 (–0.143–0.097)	–0.106 (–0.223–0.011)	0.082 (–0.035–0.199)
5	0.477 (0.357–0.597)*	0.054 (–0.065–0.074)	0.261 (0.144–0.378)*	0.128 (0.011–0.245)*
3–5	0.176 (0.057–0.296)*	–0.020 (–0.139–0.100)	0.061 (–0.056–0.178)	–0.047 (–0.163–0.070)
3	0.442 (0.322–0.562)*	0.060 (–0.060–0.179)	0.290 (0.173–0.407)*	0.110 (–0.007–0.227)
1	N/A	N/A	N/A	N/A
Other Interval	–0.011 (–0.131–0.108)	–0.011 (–0.131–0.108)	–0.013 (–0.130–0.104)	–0.005 (–0.122–0.112)
*Agreement beyond chance, P < 0.05.				

For Bard ( Table 4 Fig. 3 ), inter-rater reliability was lower in the safety-net setting (kappa = 0.148; 95% CI 0.086–0.210) only achieving slight agreement compared with fair agreement in the private hospital (kappa = 0.282; 95% CI 0.216–0.348). Furthermore, concordance was lower in the safety-net versus private hospital (46% and 54%, respectively; *P* = 0.04) with discordant early and late screening accounting for 43% and 11% of safety-net observations compared with 33% and 13% for the private hospital.

For GPT3.5 ( Table 5 ), patients with discordant versus concordant screening results were older [mean 60 years (SD 9.4) vs 57.8 (9.0); *P* = 0.01], with polypectomy performed more frequently during colonoscopy (77% vs 57%; *P* < 0.001), higher mean number of polyps [2.2 (2.4) vs 1.5 (2.0); *P* < 0.001], and mean tubular adenomas [1.1 (1.4) vs. 0.6 (1.3); *P* < 0.001]. When discordant, patients with GPT3.5 earlier versus later recommended screening intervals had more polyps found [3.8 (3.5) vs 1.6 (1.5); *P* < 0.001], but were with fewer tubular [0.6 (0.7) vs 2.3 (2.0); *P* < 0.001] or tubulovillous adenomas [0.01 (0.1) vs 0.05 (0.2); *P* = 0.03].

**Table:** **Table 5** Subanalysis of concordant and discordant results for Bard and GPT3.5.

Characteristics	Concordant with guideline panel	Discordant with guideline panel	*P* value	Discordant: Earlier screen	Discordant: Later screen	*P* value
GPT3.5	N = 328 (60.4%)	N = 215 (39.6%)		N = 156 (72.6%)	N = 59 (27.4%)	
Demographics						
Age, mean (SD)	57.8 (9.0)	60.0 (9.4)	0.01*	60.2 (9.2)	59.5 (10.2)	0.64
Gender female, N (%)	171 (52.1)	123 (57.2)	0.25	93 (59.6)	30 (50.8)	0.25
Family history of colon cancer, N (%)	18 (6.4)	21 (9.8)	0.06	12 (7.6)	9 (15.2)	0.10
Hospital safety-net, N (%)	161 (49.0)	105 (48.8)	0.96	73 (46.8)	32 (54.2)	0.33
Polypectomy performed, N (%)	186 (56.7)	165 (76.7)	< 0.001*	116 (74.3)	49 (83.0)	0.18
Total polyps found, mean (SD)	1.5 (2.0)	2.2 (2.4)	< 0.001*	1.6 (1.5)	3.8 (3.5)	< 0.001*
Polyp type						
Hyperplastic, mean (SD)	0.4 (1.0)	0.6 (1.5)	0.13	0.5 (1.0)	0.9 (2.4)	0.11
When > 0	1.7 (1.4)	1.9 (2.2)	0.48			
Tubular adenoma, mean (SD)	0.6 (1.3)	1.1 (1.4)	< 0.001*	0.6 (0.7)	2.3 (2.0)	< 0.001*
When > 0	2.1 (1.5)	2.0 (1.4)	0.74			
Tubulovillous, mean (SD)	0.04 (0.2)	0.02 (0.1)	0.22	0.01 (0.1)	0.05 (0.2)	0.03*
When > 0 (n = 16)	1.2 (0.4)	1.0 (0.0)	0.40			
Sessile serrated, mean (SD)	0.01 (0.1)	0.02 (0.2)	0.37	0.01 (0.1)	0.05 (0.3)	0.17
When > 0 (n = 8)	1.0 (0.0)	1.3 (0.5)	0.36			
Bard	N = 273 (50.0%)	N = 272 (50.0%)		N = 208 (76.5%)	N = 64 (23.5%)	
Demographics						
Age, mean (SD)	58.0 (9.3)	59.4 (9.1)	0.08	58.9 (8.9)	60.9 (9.8)	0.12
Gender female, N (%)	149 (54.6)	146 (53.7)	0.83	114 (54.8)	32 (50.0)	0.50
Family history of colon cancer, N (%)	8 (2.9)	30 (11.0)	< 0.001*	19 (9.1)	11 (17.2)	0.07
Hospital safety-net, N (%)	122 (44.7)	145 (53.3)	0.04*	116 (55.8)	29 (45.3)	0.14
Polypectomy performed, N (%)	151 (55.3)	201 (73.9)	< 0.001*	147 (70.7)	54 (84.3)	0.03*
Total polyps found, mean (SD)	1.5 (2.2)	2.0 (2.2)	0.004*	1.6 (1.7)	3.5 (2.9)	< 0.001*
Polyp type						
Hyperplastic, mean (SD)	0.4 (1.4)	0.6 (1.0)	0.17	0.6 (1.0)	0.6 (1.2)	0.81
When > 0	2.0 (2.5)	1.7 (1.1)	0.31			
Tubular Adenoma, mean (SD)	0.8 (1.3)	0.9 (1.4)	0.19	0.5 (0.8)	2.3 (1.9)	< 0.001*
When > 0	2.0 (1.5)	2.0 (1.4)	0.55			
Tubulovillous, mean (SD)	0.3 (0.2)	0.3 (0.2)	0.82	0 (0.1)	0.1 (0.4)	< 0.001*
When > 0 (n = 15)	1.1 (0.4)	1.1 (0.4)	0.93			
Sessile serrated, mean (SD)	0.01 (0.1)	0.02 (0.2)	0.36	0.01 (0.1)	0.05 (0.3)	0.18
When > 0 (n = 8)	1.0 (0.0)	1.2 (0.4)	0.48			
SD, standard deviation. *Agreement beyond chance, P < 0.05.						

**Fig. 4:**
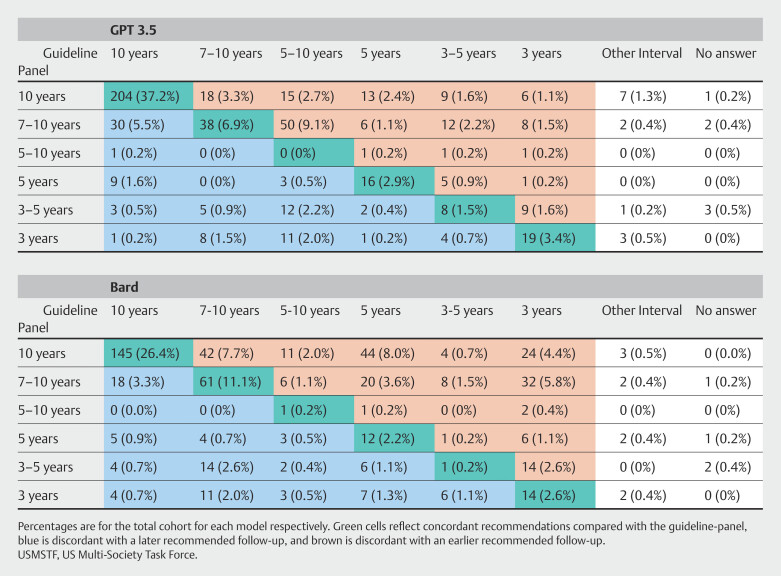
Comparison of ChatGPT3.5, Bard, and USMSTF Panel recommendations for colonoscopy follow-up intervals.

For Bard ( Table 5 ), patients with discordant versus concordant screening results were more likely to have a family history of colon cancer (11% vs 3%; *P* < 0.001), more frequent polypectomy performed during colonoscopy (74% vs 55%; *P* < 0.001), more polyps found [mean 2.0 (SD 2.2) vs 1.5 (2.2); *P* = 0.004], and more likely to be from the private hospital (54% vs. 45%; *P* = 0.04). When discordant, patients with Bard earlier versus later recommended screening intervals were less likely to have a polypectomy performed during the colonoscopy (71% vs 84%, *P* = 0.03), had fewer polyps found (mean (SD): 1.6 (1.7) vs 3.5 (2.9), *P* < 0.001) and were with fewer tubular (mean (SD): 0.5(0.8) vs 2.3 (1.9), *P* < 0.001) and tubulovillous (mean (SD): 0 (0.1) vs 0.1(0.4), *P* < 0.001) adenomas. A visual comparison of concordance between the LLMs and the guideline panel is demonstrated in Fig. 4 .

## Discussion

This study compared performance of two freely available LLMs, ChatGPT3.5 turbo by OpenAI and Bard by Google, in making follow-up interval recommendations after colonoscopy. The potential for LLMs to serve as a second reader to ensure uniformity of colonoscopy results and propose intervals for follow-up is noteworthy, particularly considering the rising prominence of AI in healthcare 10 12 24 . Our principal findings revealed that ChatGPT3.5 outperformed Bard in alignment with guideline-panel recommendations. The difference in agreement between the two models was particularly pronounced for the safety-net hospital cohort, where GPT3.5 was found to have better agreement in its recommendations. The similar performance of GPT3.5 between the two hospitals underscores its utility, irrespective of hospital type, and underscores its potential ability as a secondary viewer to validate recommendations.

Furthermore, a detailed analysis revealed limitations in prediction tendencies of both LLMs. We discovered that both models tended to deviate from the recommended intervals based on specific clinical features. For instance, GPT3.5 was inclined to suggest earlier screening for patients with a family history of colon cancer and more detected polyps, potentially leading to an unnecessary increase in costs, but proposed later intervals when more tubular or tubulovillous adenomas were identified, a potentially problematic result given the known risk from advanced adenomas 29 . Bard recommendations were similarly more likely to be discordant dependent on patient age, family history, and presence of specific adenomas and recommended earlier screening if a polypectomy was performed. Similar to GPT3.5, it also recommended later screening if tubular or tubulovillous adenomas were found. However, unlike GPT3.5, Bard was more likely to have discordant results in the safety-net hospital compared with the private hospital.

The observed variations underscore the importance of understanding clinical nuances that might influence LLM decisions. Such insights are invaluable, especially when considering integration of these LLMs into clinical workflows as auxiliary aids to human experts.

Differences in outcomes between the two hospitals are also an important consideration. Disparities between private and safety-net systems are often rooted in systemic issues such as resource allocation, socioeconomic determinants, and varied patient populations, and have been well-documented in the literature 30 31 . Introduction of LLMs like GPT3.5 and Bard might help mitigate such disparities. In particular, as we observed, the similar performance of GPT3.5 across two distinct hospital settings suggests that such models could potentially ensure uniformity in recommendations and reduce hospital-specific biases.

This study, however, has several limitations. Recommendations from both LLMs diverged from guidelines established by the endoscopist panel and had relatively low accuracy. These deviations are likely because the LLMs are not specifically trained on colonoscopy data or tailored for gastroenterology. Rather, their vast training on general data enables them to handle a wide array of queries, which includes free-text data entry. This underscores the adaptability and versatility of such models, but also raises questions about their depth of specialization in niche medical domains.

GPT3.5 and Bard were particularly chosen for this study because they are freely available, which is particularly important for safety-net hospitals that may lack resources to access more sophisticated, paid AI programs. However, we also recognize the availability of newer, paid models that promise better accuracy and training on larger sets of data, which we previously demonstrated with GPT4 16 . Unlike GPT-3.5, GPT-4 was trained on a larger and more diverse dataset, and its architecture was optimized for complex reasoning, which may contribute to better accuracy in nuanced applications, such as colonoscopy interval recommendations. Recent studies support this performance gap: GPT-4 has achieved significantly higher scores on medical licensing exams compared with GPT-3.5, reflecting its superior ability to interpret and apply medical guidelines accurately 32 . Moreover, GPT-4 has shown improved accuracy in general-purpose tasks by capturing context more effectively, as observed in the OpenAI benchmark test 33 . These advancements suggest that GPT-4 is inherently better equipped to handle the clinical decision-making processes. Although our choice of GPT-3.5 aligns with the needs of safety-net hospitals, we recognize that further studies using GPT-4 could provide valuable insights into the impact of advanced model architectures on clinical outcomes.

Although LLMs promise enhanced uniformity and efficiency, relying solely on their recommendations without clinician oversight is premature. Integration of these tools into clinical practice, especially when combined with human expertise, may enhance patient care and help mitigate disparities in healthcare settings. Given these considerations and the success of previous machine-learning studies that have utilized pre-training with existing data 6 7 , pre-training of LLMs is a logical next step in utilizing these models for future applications.

At our safety-net hospital, CRC screening is predominantly conducted using annual fecal immunohistochemistry (FIT) testing due to resource limitations. Patients are referred for colonoscopy only following a positive FIT result. This practice reflects the real-world approach to screening in resource-limited settings and shaped the major composition of our safety-net population. To minimize inclusion of younger, symptomatic patients, we restricted our analysis to individuals aged 45 and older, aligning with current guidelines for average-risk colorectal cancer screening.

In our analysis, the average number of polyps detected per patient was higher in the safety-net hospital compared with the private hospital (2.1 vs 1.5). However, the number of tubular adenomas detected per patient did not differ significantly between the two settings. This finding suggests that although total polyp burden may vary by practice setting, it is unclear if this clinical nuance had an influence on the results and raises the question of whether the increased overall polyp burden might have influenced Bard accuracy. Additional factors, such as differences in clinical documentation or contextual variability between safety-net and private hospitals, may also have contributed to the observed disparity. Further investigation is warranted to better understand how these variables impact performance of LLMs in diverse clinical environments.

In cases for which the recommended surveillance interval is a range (e.g., 7–10 years or 3–5 years), lower accuracy observed in LLMs may be attributed to challenges in interpreting nuanced clinical features, such as the number and type of adenomas. For example, per current guidelines, a 7- to 10-year interval is recommended when one to two adenomas < 10 mm are identified, whereas a 3- to 5-year interval applies for three to four adenomas < 10 mm or certain advanced findings (e.g., 3 to 4 sessile serrated polyps [SSPs] or a hyperplastic polyp > 10 mm). As shown in Table 5 , GPT-3.5 exhibited higher discordance in cases with a greater number of tubular adenomas, suggesting that LLMs may struggle to parse the complexity of such scenarios, contributing to this observed trend.

An interesting observation in the LLM-generated outputs is inclusion of disclaimers such as, “Your doctor may recommend a different colonoscopy interval,” even when the prompt does not simulate a patient inquiry. This likely reflects a built-in mechanism to mitigate risk of providing explicit medical advice, consistent with the terms of service for these models, which explicitly caution against using their outputs as definitive clinical recommendations 17 . This hedging behavior may also serve to reduce liability, underscoring the need for human oversight in interpreting LLM-generated guidance.

The relatively low number of patients with SSPs in our study warrants explanation. This finding is likely attributable to our exclusion criteria, specifically exclusion of patients referred for advanced therapeutic procedures such as endoscopic mucosal resection. SSPs, particularly larger ones, often require advanced endoscopic techniques for removal due to their flat morphology and potential for submucosal invasion. By excluding patients referred for endoscopic mucosal resection and similar procedures, we may have inadvertently filtered out a significant portion of SSP cases, especially those of larger size or more complex presentation. This limitation should be considered when interpreting our results, because it may underrepresent the true prevalence and characteristics of SSPs in the broader patient population undergoing screening colonoscopy. Moreover, absence of SSPs at the safety-net hospital is noteworthy. This hospital primarily serves a Latino population, and there are limited data on prevalence of SSPs within this demographic group, with some studies noting a significantly lower rate of SSPs in this demographic 34 35 . Lack of SSPs in this subset of our study population may reflect genuine epidemiological differences, or it could be due to other factors such as variations in detection rates or referral patterns.

Another important limitation of this study is variability in documentation formats and quality between the two institutions. At the safety-net hospital, colonoscopy reports and history of present illness notes follow a standardized, structured format, whereas the private hospital utilizes free-text documentation, which displayed varying consistency depending on the note author. In addition, the two institutions use different EMR systems, each with unique input formats. These differences in documentation structure and quality may have influenced accuracy of AI predictions, introducing potential bias when comparing outcomes between the safety-net and private hospitals.

## Conclusions

This study underscores limitations of using freely available LLMs in diverse healthcare settings, emphasizing the need for careful integration of these tools in clinical practice. Although previous studies utilizing paid models have demonstrated better results, this is the first study that has looked at the utility of and potential of freely available LLMs 16 24 . The potential of freely available LLMs to offer uniformity in recommendations is significant; however, the low accuracy as noted in our study excludes their use as the sole agent in recommending colonoscopy interval recommendations. As AI continues to evolve, its thoughtful application in healthcare promises to bridge disparities and augment patient care; however, significant work must be done before it has reached its potential for implementation into patient care.

## Supplementary Material

Supplementary Material Zusatzmaterial

## References

[1] Siegel RL Miller KD Jemal A Cancer statistics, 2020 CA Cancer J Clin 2020 70 7 30 10.3322/caac.21590 31912902

[2] Nishihara R Wu K Lochhead P Long-term colorectal-cancer incidence and mortality after lower endoscopy N Engl J Med 2013 369 1095 1105 10.1056/NEJMoa1301969 24047059 PMC3840160

[3] US Preventive Services Task Force Davidson KW Barry MJ Screening for colorectal cancer: US Preventive Services Task Force recommendation statement JAMA 2021 325 1965 34003218 10.1001/jama.2021.6238

[4] Kröner PT Engels MM Glicksberg BS Artificial intelligence in gastroenterology: A state-of-the-art review World J Gastroenterol 2021 27 6794 6824 10.3748/wjg.v27.i40.6794 34790008 PMC8567482

[5] Hassan C Wallace MB Sharma P New artificial intelligence system: first validation study versus experienced endoscopists for colorectal polyp detection Gut 2020 69 799 800 10.1136/gutjnl-2019-319914 31615835

[6] Karwa A Patell R Parthasarathy G Development of an automated algorithm to generate guideline-based recommendations for follow-up colonoscopy Clin Gastroenterol Hepatol 2020 18 2038 20450 31622739 10.1016/j.cgh.2019.10.013

[7] Peterson E May FP Kachikian O Automated identification and assignment of colonoscopy surveillance recommendations for individuals with colorectal polyps Gastrointest Endosc 2021 94 978 987 34087201 10.1016/j.gie.2021.05.036

[8] Sharma P Parasa S ChatGPT and large language models in gastroenterology Nat Rev Gastroenterol Hepatol 2023 20 481 482 10.1038/s41575-023-00799-8 37253794

[9] Pugliese N Wong VW-S Schattenberg JM Accuracy, reliability and comprehensiveness of ChatGPT generated medical responses for patients with NAFLD Clin Gastroenterol Hepatol 2023 22 886 889 37716618 10.1016/j.cgh.2023.08.033

[10] Lee T-C Staller K Botoman V ChatGPT answers common patient questions about colonoscopy Gastroenterology 2023 165 509 5.11E9 37150470 10.1053/j.gastro.2023.04.033

[11] Yeo YH Samaan JS Ng WH Assessing the performance of ChatGPT in answering questions regarding cirrhosis and hepatocellular carcinoma Clin Mol Hepatol 2023 29 721 732 36946005 10.3350/cmh.2023.0089 PMC10366809

[12] Levartovsky A Ben-Horin S Kopylov U Towards AI-augmented clinical decision-making: An examination of ChatGPT’s Utility in acute ulcerative colitis presentations Am J Gastroenterol 2023 118 2283 2289 10.14309/ajg.0000000000002483 37611254

[13] Uche-Anya E Anyane-Yeboa A Berzin TM Artificial intelligence in gastroenterology and hepatology: how to advance clinical practice while ensuring health equity Gut 2022 71 1909 1915 35688612 10.1136/gutjnl-2021-326271 PMC10323754

[14] Chen IY Szolovits P Ghassemi M Can AI help reduce disparities in general medical and mental health care? AMA J Ethics 2019 21 E167 E179 10.1001/amajethics.2019.167 30794127

[15] Fedewa SA Flanders WD Ward KC Racial and ethnic disparities in interval colorectal cancer incidence: A population-based cohort study Ann Intern Med 2017 166 857 866 10.7326/M16-1154 28531909 PMC5897770

[16] Chang PW Amini MM Davis RO ChatGPT4 outperforms endoscopists for determination of post-colonoscopy re-screening and surveillance recommendations Clin Gastroenterol Hepatol 2024 22 1917 1925 38729387 10.1016/j.cgh.2024.04.022

[17] OpenAI Platform https://platform.openai.com

[18] Pichai S. An important next step on our AI journey. Google 2023 https://blog.google/technology/ai/bard-google-ai-search-updates/

[19] Brown T Mann B Ryder N Language models are few-shot learners Advances in neural information processing systems 2020 33 1877 1901

[20] Google AI PaLM 2. arXiv:2005.14165 https://arxiv.org/abs/2005.14165

[21] Anil R, Dai AM, Firat O et al. PaLM 2 Technical Report 2023 https://doi.org/10.48550/arXiv.2305.10403

[22] Elias J. Google’s newest A.I. model uses nearly five times more text data for training than its predecessor. CNBC 2023 https://www.cnbc.com/2023/05/16/googles-palm-2-uses-nearly-five-times-more-text-data-than-predecessor.html

[23] Gupta S Lieberman D Anderson JC Recommendations for follow-up after colonoscopy and polypectomy: A consensus update by the US Multi-Society Task Force on Colorectal Cancer Gastrointest Endosc 2020 91 463 4.85E7 32044106 10.1016/j.gie.2020.01.014 PMC7389642

[24] Gorelik Y Ghersin I Maza I Harnessing language models for streamlined postcolonoscopy patient management: a novel approach Gastrointest Endosc 2023 98 639 6.41E6 37385548 10.1016/j.gie.2023.06.025

[25] Lahat A Shachar E Avidan B Evaluating the use of large language model in identifying top research questions in gastroenterology Sci Rep 2023 13 4164 10.1038/s41598-023-31412-2 36914821 PMC10011374

[26] Khanna R Nelson SA Feagan BG Endoscopic scoring indices for evaluation of disease activity in Crohn’s disease Cochrane Database Syst Rev 2016 2016 CD010642 10.1002/14651858.CD010642.pub2 PMC7079710 27501379

[27] Zorron Cheng Tao Pu L Chiam KH Yamamura T Narrow-band imaging for scar (NBI-SCAR) classification: from conception to multicenter validation Gastrointest Endosc 2020 91 1146 1.154E8 10.1016/j.gie.2019.08.036 31494134

[28] Landis JR Koch GG The measurement of observer agreement for categorical data Biometrics 1977 33 159 843571

[29] Winawer SJ Zauber AG The advanced adenoma as the primary target of screening Gastrointest Endosc Clin North Am 2002 12 1 9 10.1016/s1052-5157(03)00053-9 11916153

[30] Liss DT Baker DW Understanding current racial/ethnic disparities in colorectal cancer screening in the United States: the contribution of socioeconomic status and access to care Am J Prev Med 2014 46 228 236 10.1016/j.amepre.2013.10.023 24512861

[31] Habchi KM Weinberg RY White RS How the use of standardized protocols may paradoxically worsen disparities for safety-net hospitals J Comp Eff Res 2022 11 65 66 10.2217/cer-2021-0289 34879744

[32] Meyer A Riese J Streichert T Comparison of the performance of GPT-3.5 and GPT-4 with that of medical students on the written German medical licensing examination: Observational study JMIR Medical Education 2024 10 e50965 10.2196/50965 PMC10884900 38329802

[33] GPT-4 https://openai.com/index/gpt-4/

[34] Ali MF Grewal J Karnes W Screening colonoscopy polyp, adenoma and sessile serrated adenoma detection rate differences in Hispanics and Whites in age-matched cohorts Am J Gastroenterol 2018 113 S62

[35] Edwardson N Adsul P Gonzalez Z Sessile serrated lesion detection rates continue to increase: 2008–2020 Endosc Int Open 2023 11 E107 E116 10.1055/a-1990-0509 36712908 PMC9879655

