# Supplementary material for: Comparing ChatGPT3.5 and Bard recommendations for colonoscopy intervals: Bridging the gap in healthcare settings
Source: Endosc Int Open. 2025 Jun 17;13:a25865912. doi: 10.1055/a-2586-5912 (PMC12223955; doi:10.1055/a-2586-5912)
Supplement: Supplementary file 1 — Supplementary Material [file 10-1055-a-2586-5912_25959988.pdf]

Supplementary Table 1 Sample Verbatim input and output into ChatGPT3.5 and Bard with associated recommended intervals by ChatGPT3.5, Bard, and AGA Guideline-based panel.

	LLM input	ChatGPT3.5 output	Bard output	ChatGPT 3.5 reccomended interval	Bard	AGA Guideline-based panel
Example 1. Correct, all answers aligned	"Based on this, brief patient data, family history, colonoscopy report and pathology data, what would be recommended next colonoscopy interval using the AGA 2020 guidelines? Please do not summarize report and please include recommend interval.<br>52 year old male who presents for a screening colonoscopy<br>There is not a family history of 1st degree relative with colon cancer.<br>The terminal ileum appeared normal.	"Based on the provided patient data and the AGA (American Gastroenterological Association) 2020 guidelines for colonoscopy screening intervals, the recommended next colonoscopy interval for this 52-year-old male without a family history of first-degree relatives with colon cancer would be as follows:<br><br>For a patient with no significant findings during the screening colonoscopy, such as polyps or	Based on the AGA 2020 guidelines, the recommended next colonoscopy interval for a 52-year-old male with a negative colonoscopy and no family history of colon cancer is 10 years.	10 years	10 years	10 years

	- Scattered small-mouthed diverticula were found in the sigmoid colon.<br>no path"	abnormal pathology, and in the absence of a family history of colon cancer in first-degree relatives, the recommended colonoscopy interval, according to AGA 2020 guidelines, would typically be:<br><br>Next colonoscopy in 10 years.<br><br>Therefore, it is advisable for this patient to undergo their next colonoscopy in 10 years from the date of the initial screening colonoscopy, assuming no new symptoms or risk factors develop in the interim. However, it's essential to follow up with the patient's healthcare provider for personalized recommendations	

		and to discuss any potential changes in the recommended interval based on their specific health and risk factors."				
Example 2. ChatGPT 3.5 concordant, Bard discordant	"Based on this, brief patient data, family history, colonoscopy report and pathology data, what would be recommended next colonoscopy interval using the AGA 2020 guidelines? Please do not summarize report and please include recommend interval.<br>71 year old with h/o TA in 2017<br>There is not a family history of 1st degree relative with colon cancer.<br>The terminal ileum appeared normal.<br>- Two sessile polyps were found in the cecum. The polyps were 1 to 2 mm in	"The recommended colonoscopy interval for this patient, based on the provided patient data, family history, colonoscopy report, and pathology data, using the AGA 2020 guidelines, would typically be as follows:<br><br>1. **Age**: The patient is 71 years old.<br><br>2. **Personal History of Tubular Adenomas (TA) in 2017**: This is an important factor. According to the AGA 2020 guidelines, if a patient has a history of low-risk	"Based on the AGA 2020 guidelines, the recommended next colonoscopy interval for this patient is **7-10 years**.<br><br>**Rationale:**<br><br>* The patient is 71 years old, which is within the recommended age range for colorectal cancer screening (50-75 years old).<br>* The patient has a history of tubular adenomas, which are considered to be low-risk polyps.<br>* The patient's most recent colonoscopy showed two sessile tubular adenomas in the cecum, a sessile tubular adenoma in the	3-5 years	7-10 years	3-5 years

	size. These polyps were removed with a cold biopsy forceps. Resection and retrieval were complete. Estimated blood loss: none.<br>- A 2 mm polyp was found in the descending colon. The polyp was sessile. The polyp was removed with a cold biopsy forceps. Resection and retrieval were complete. Estimated blood loss: none.<br>- Two flat polyps were found in the rectum. The polyps were 1 to 2 mm in size. These polyps were removed with a cold biopsy forceps. Resection and retrieval were complete.<br>- Multiple small and large-mouthed	adenomas (like tubular adenomas) and these adenomas were removed completely, the recommended interval for follow-up colonoscopy is usually 5 to 10 years. Given the patient's age and history, a more conservative approach might lean towards a shorter interval.<br><br>3. **Family History**: There is no family history of first-degree relatives with colon cancer. Family history is typically considered when assessing colonoscopy intervals, but in this case, it does not impact the recommendation.	descending colon, and two hyperplastic polyps in the rectum.<br>* The patient has no family history of colorectal cancer.<br><br>The AGA 2020 guidelines recommend a repeat colonoscopy interval of 7-10 years for patients with 1-2 small tubular adenomas. The patient in this case has 2 small tubular adenomas, so the recommended interval is 7-10 years.<br><br>It is important to note that the AGA 2020 guidelines are just general recommendations. The specific colonoscopy interval for a patient should be individualized based on their individual risk factors. For example, a patient with a strong family history of colorectal cancer may	

	diverticula were found in the entire colon. More pronounced in the left colon<br>- Internal hemorrhoids were found during retroflexion. The hemorrhoids were small.<br>A. CECAL POLYP X2:<br>- Tubular adenomata<br><br>B. DESCENDING COLON POLYP:<br>- Tubular adenoma<br>- Focal minimal melanosis coli<br><br>C. RECTAL POLYP X2:<br>- Hyperplastic polyp<br>"	4. **Findings from Colonoscopy and Pathology**:<br>- Cecal Polyps (x2): Tubular adenomata.<br>- Descending Colon Polyp: Tubular adenoma with minimal melanosis coli.<br>- Rectal Polyps (x2): Hyperplastic polyps.<br>- Other Findings: Multiple diverticula throughout the colon, more pronounced in the left colon, and small internal hemorrhoids.<br><br>Given the patient's age and the presence of tubular adenomas in the cecum and descending colon, it is advisable to have a follow-up colonoscopy within 3 to 5 years. This is a	need to have their colonoscopy more frequently.<br><br>**Recommendation:**<br><br>The patient should have their next colonoscopy in 7-10 years."	

	more conservative approach, considering both the age of the patient and the adenomatous polyps found during the colonoscopy. However, the final recommendation should be made in consultation with the patient's healthcare provider, taking into account their specific medical history and risk factors.<br><br>Please note that individual patient recommendations may vary, and it's essential for the patient to discuss the appropriate colonoscopy interval with their healthcare provider, who can provide personalized guidance based on all available information."	

Example 3. GPT3.5 discordant, Bard concordant	"Based on this, brief patient data, family history, colonoscopy report and pathology data, what would be recommended next colonoscopy interval using the AGA 2020 guidelines? Please do not summarize report and please include recommend interval.<br>64M with history of HLD, HTN, T2DM referred for EGD+colonoscopy.<br><br>EGD for re-evaluation of prior gastric ulceration. Colonoscopy for surveillance given single diminutive TA on colonoscopy 11/2016 (7 years ago).<br><br>Labs 3/2023 with Hgb 12.9/MCV 87/RDW 14.4%/iron 106/ferritin 23. Hgb	"Based on the provided patient data, family history, colonoscopy report, and pathology data, and considering the AGA (American Gastroenterological Association) 2020 guidelines, here is the recommended next colonoscopy interval:<br><br>1. The patient is a 64-year-old male with a history of hyperlipidemia (HLD), hypertension (HTN), and type 2 diabetes mellitus (T2DM).<br><br>2. The reason for the colonoscopy was surveillance due to a single diminutive tubular adenoma (TA) found on a colonoscopy performed in November 2016	"**Next colonoscopy interval using the AGA 2020 guidelines:**<br><br>**7-10 years**<br><br>**Rationale:**<br><br>* The patient had a single diminutive (≤5 mm) tubular adenoma on colonoscopy 7 years ago.<br>* The patient has no other high-risk features for colorectal cancer, such as a family history of first-degree relative with colon cancer or multiple or large adenomas.<br><br>The AGA 2020 guidelines recommend a colonoscopy interval of 7-10 years for patients with a single diminutive tubular adenoma. This is based on evidence that the risk of developing advanced neoplasia (adenomas ≥10 mm or	3-5 years	7-10 years	7-10 years

	has been stable in high 12-low 13 range since 2015. There is not a family history of 1st degree relative with colon cancer.<br>Findings:<br><br>Excavated lesions Multiple diverticula with mixed openings were seen in the whole colon majority of which were in the SC and DC. Diverticulosis appeared to be of moderate severity.<br><br>Protruding lesions Medium grade/stage II internal hemorrhoids were noted.<br><br>A single sessile 4 mm polyp of benign appearance was found in the transverse	(approximately 7 years ago).<br><br>3. There is no family history of a first-degree relative with colon cancer.<br><br>4. The recent labs from March 2023 showed a stable hemoglobin (Hgb) level in the high 12 to low 13 range.<br><br>5. The recent colonoscopy findings include:<br>- Moderate severity diverticulosis throughout the whole colon.<br>- Medium-grade (stage II) internal hemorrhoids.<br>- A single sessile 4 mm polyp with a benign appearance in the transverse colon, which was completely removed	adenomas with high-grade dysplasia) or colorectal cancer within 10 years of removing a single diminutive tubular adenoma is low.<br><br>However, it is important to note that these are general guidelines. Your doctor may recommend a different colonoscopy interval based on your individual risk factors. For example, if you have other risk factors for colorectal cancer, such as a family history of the disease, your doctor may recommend more frequent colonoscopies.<br><br>It is also important to discuss your colonoscopy interval with your doctor, especially if you have any concerns."

	colon.A single-piece polypectomy was performed using a cold forceps in the transverse colon. The polyp was completely removed and retrieved.<br>Final Pathologic Diagnosis<br>A. Stomach antrum, body, incisura, fundus:<br>- Gastric oxyntic and antral mucosa showing no significant lesion<br>- No Helicobacter pylori identified<br><br>B. TC polyp:<br>- Tubular adenoma"	and retrieved during the procedure.<br><br>Based on these findings and the AGA 2020 guidelines, the recommended next colonoscopy interval would be as follows:<br><br>1. Given the presence of a tubular adenoma (even though it was benign) and the patient's age of 64, it is generally recommended to repeat the colonoscopy in 3 to 5 years for surveillance. This is to monitor for the development of new polyps or adenomas.<br><br>2. In this case, it is reasonable to consider scheduling the next colonoscopy in 3 years (assuming there are no other

	clinical factors that would suggest a different interval).<br><br>Please note that individual patient recommendations may vary based on the specific clinical circumstances and the discretion of the treating physician. It's essential for the patient to discuss the recommended interval with their healthcare provider, taking into account their overall health and risk factors."	

AGA, American Gastroenterological Association; DC, descending colon; EGD, esophagogastroduodenoscopy; Hgb, hemoglobin; HLD, hyperlipidemia; HTN, hypertension; LLM, large language model; MCV, mean corpuscular volume; RDW, red cell distribution width; TA, tubular adenoma; T2DM, type 2 diabetes mellitus.
